# Integrated bioinformatics analysis for differentially expressed genes and signaling pathways identification in gastric cancer

**DOI:** 10.7150/ijms.47339

**Published:** 2021-01-01

**Authors:** ChenChen Yang, Aifeng Gong

**Affiliations:** 1 Department of Emergency, The Affiliated Huaian No. 1 People's Hospital of Nanjing Medical University, Huai'an 223300, Jiangsu, China.; 2 Department of Gerontology, The Affiliated Huaian No. 1 People's Hospital of Nanjing Medical University, Huai'an, 223300, Jiangsu, China.

**Keywords:** gastric cancer, GEO data, differentially expressed genes, integrated bioinformatics

## Abstract

**Background:** Gastric cancer (GC) has a high mortality rate in cancer-related deaths worldwide. Currently, the pathogenesis of gastric cancer progression remains unclear. Here, we identified several vital candidate genes related to gastric cancer development and revealed the potential pathogenic mechanisms using integrated bioinformatics analysis.

**Methods:** Two microarray datasets from Gene Expression Omnibus (GEO) database integrated. Limma package was used to analyze differentially expressed genes (DEGs) between GC and matched normal specimens. DAVID was utilized to conduct Gene ontology (GO) and KEGG enrichment analysis. The relative expression of OLFM4, IGF2BP3, CLDN1 and MMP1were analyzed based on TCGA database provided by UALCAN. Western blot and quantitative real time PCR assay were performed to determine the protein and mRNA levels of OLFM4, IGF2BP3, CLDN1 and MMP1 in GC tissues and cell lines, respectively.

**Results:** We downloaded the expression profiles of GSE103236 and GSE118897 from the Gene Expression Omnibus (GEO) database. Two integrated microarray datasets were used to obtain differentially expressed genes (DEGs), and bioinformatics methods were used for in-depth analysis. After gene ontology (GO) and Kyoto Encyclopedia of Genes and Genomes (KEGG) pathway enrichments analysis, we identified 61 DEGs in common, of which the expression of 34 genes were elevated and 27 genes were decreased. GO analysis displayed that the biological functions of DEGs mainly focused on negative regulation of growth, fatty acid binding, cellular response to zinc ion and calcium-independent cell-cell adhesion. KEGG pathway analysis demonstrated that these DEGs mainly related to the Wnt and tumor signaling pathway. Interestingly, we found 4 genes were most significantly upregulated in the DEGs, which were OLFM4, IGF2BP3, CLDN1 and MMP1. Then, we confirmed the upregulation of these genes in STAD based on sample types. In the final, western blot and qRT-PCR assay were performed to determine the protein and mRNA levels of OLFM4, IGF2BP3, CLDN1 and MMP1 in GC tissues and cell lines.

**Conclusion:** In our study, using integrated bioinformatics to screen DEGs in gastric cancer could benefit us for understanding the pathogenic mechanism underlying gastric cancer progression. Meanwhile, we also identified four significantly upregulated genes in DEGs from both two datasets, which might be used as the biomarkers for early diagnosis and prevention of gastric cancer.

## Introduction

Gastric cancer (GC) is one of the solid tumors with a higher morality worldwide, and the 5-year survival rate for GC patients is about 20% globally 1, 2. Investigation showed that initially diagnosed at advanced stage should be responsible for poor 5-year survival of GC 3-5. Despite numerous studies have partly revealed the molecular mechanisms of GC, and the emerging therapeutic options have been implemented 6, 7, there also have patients with GC could hardly respond to existing molecularly targeted agents. To date, the promising novel diagnostic and prognostic biomarkers of GC still remain unclear. Hence, there has an urgent demand for identifying the sensitive and specific biomarkers of GC.

Recently, gastric gene expression profiles have been investigated in many studies, and thousands of differentially expressed genes (DEGs) have been screened, which might be related to the GC progression 8-12. Due to specimens that were collected from different backgrounds and analyzed using different technological detection platforms, it is discrepant in the identification of significantly expressed mRNAs among each independent experiment. Thus, an unbiased approach should be performed to integrate the results from single-cohort study. The microarray and high throughput sequencing technologies have been improved in recent years and used to identify the promising candidate biomarkers for diagnostic application of cancer treatment during cancer development 13. In order to overcome the inconsistent results, integrated bioinformatics methods have been applied to uncover the valuable biological information in cancer research 14, 15.

In our study, the expression profiles of GSE103236 and GSE118897 from GEO database were downloaded and further analyzed. The GO pathway enrichment analysis of DEGs was conducted on DAVID (https://david.ncifcrf.gov/) and the KEGG pathways (http://kobas.cbi.pku.edu.cn/). After GO analysis, we verified the expressions of OLFM4, IGF2BP3, CLDN1 and MMP1 were significantly elevated in the DEGs using western blotting and qRT-PCR assay. In conclusion, integrated bioinformatics methods help us to screen DEGs and pathways in gastric cancer and understand the pathogenic mechanism underlying gastric cancer development. Moreover, we also revealed four significantly upregulated genes in DEGs, which might be the diagnostic and prognostic biomarkers of GC.

## Methods

### Specimen collection

The gastric cancer (GC) tissues and corresponding adjacent gastric tissues were obtained from “The Affiliated Huaian No. 1 People's Hospital of Nanjing Medical University” between Jan. 2012 and Jan. 2013. 30 pairs of tissues in total were analyzed in this study. No systemic treatment of chemotherapy or radiotherapy was conducted in these patients before surgery. All of patients had got the written informed consent before tissue collection. The study was approved by the ethics committee of “The Affiliated Huaian No. 1 People's Hospital of Nanjing Medical University”. All samples were stored at -80 °C until use.

### Cell culture

GC cell lines NCI-N87, SNU-1 and MGC80-3 were cultured in RPMI 1640 Medium (Gibco, 31800022) with 10% heat‐inactivated fetal bovine serum (FBS). GC cell lines KATO III and SNU-5 were cultured in Iscove's Modified Dulbecco's Medium (IMDM) (Invitrogen, 31980-030) with 20% heat-inactivated FBS. Human fibroblast cells Hs 738.St/Int were cultured in dulbecco's modified eagle medium (DMEM) (Invitrogen, 11960-044) with 10% heat‐inactivated FBS. All cultured cells were incubated in a humidified atmosphere containing 5% CO2 at 37 °C. We purchased all used cell lines from the Institute of Biochemistry and Cell Biology at the Chinese Academy of Science (Shanghai, China).

### Microarray data sets

Gene Expression Omnibus (GEO) (https://www.ncbi.nlm.nih.gov/geo/) is a publicly available genomics database, which could be queried for all data sets. We downloaded two data sets of GC, which were the gene expression profiles of GSE103236 and GSE118897, from GEO. The selected datasets in accordance with the following criteria and reason: (1) The dataset was uploaded between 2017.2.1 to 2020.2.1. (2) The GC tissue samples were employed, and the adjacent normal tissues were used as control. It is known that *H.pylori* infection is one of the most important factors for GC studies. However, we did not find the *H.pylori* data in the selected datasets. This criteria, including GC tissues and normal tissues in one dataset, would help keep the balance of *H.pylori* infection status between tumor and control tissue samples. (3) Studies had detail information on technology and platform, which were utilized for datasets analysis.

### Integration of microarray data and screening for DEGs

Generally, the variability of data is mainly from heterogeneity and potential variables. The data sets we analyzed in this study were based on different platforms, while the samples were handled in different groups. Therefore, in order to generated more reliable results, we performed the normalization and base-2 logarithm conversion for the matrix data of each GEO dataset using the limma package in R software 16. Furthermore, we performed gene differential analysis by comparing tumor tissues with normal tissues. |logFC| >1, *P*-value < 0.05 and adjusted *P*-value < 0.05 were considered to be statistically significant for the DEGs. We kept a list of integrated misalignment genes for subsequent analysis.

### GO and KEGG pathway enrichment analyses of DEGs

The DAVID database (https://david.ncifcrf.gov/) is an important website for high-throughput gene function analysis. Based on the DAVID database, we analyzed and annotated the functional and pathway enrichment of candidate genes. A DAVID online tool on the screened DEGs was used to conduct the GO annotations. For KEGG pathway analysis, the KOBAS database (available online: http://kobas.cbi.pku.edu.cn/) was used. In our study, the DEGs determined from integrated microarray gastric cancer data were analyzed and *P*-value <0.05 was considered to have statistical significance.

### UALCAN

UALCAN (http://ualcan.path.uab.edu) is a comprehensive website based on level 3 RNA-seq and clinical data from 31 cancer types in The Cancer Genome Atlas (TCGA) database (https://cancergenome.nih.gov/). Researchers were allowed to analyze the relative expression of interested genes across tumor and normal samples and relative clinicopathologic parameters from web resources provided by UALCAN. In this study, 415 STAD samples and 34 matched adjacent normal samples were obtained from The Cancer Genome Atlas (TCGA).

### Western blot assay

Total proteins from cultured cells were lysed in the RIPA buffer (Beyotime, China) and quantified. After SDS‐PAGE assay, we transferred the proteins onto polyvinylidene fluoride (PVDF) membranes followed by 5% nonfat milk blocked for 1 hour (h). Next, we incubated the membranes with primary antibodies overnight at 4 °C and then washed using phosphate buffered saline supplemented with Tween 20 (PBST). We subsequently incubated the membranes with secondary antibodies at room temperature for 2 h. In the final, we took the protein bands on membranes into visualization using an enhanced chemiluminescence (ECL) detection system (Thermo Fisher Scientific, USA). The used primary and secondary antibodies were listed as follows: rabbit anti-OLFM4 antibody (1:2000, Abcam, ab105861), rabbit anti- IGF2BP3 antibody (HRP) (1:1500, Abcam, ab208869), rabbit anti-CLDN1 antibody (1:2000, Abcam, ab180158), rabbit anti-MMP1 antibody (1:2000, Abcam, ab38929), rabbit anti-GAPDH (1:3000, Abcam, ab181603) and goat anti-rabbit IgG H&L (HRP) (1:3000, Abcam, ab205718). We used GAPDH as the endogenous control.

### Quantitative real-time polymerase chain reaction (qRT-PCR)

Extraction of Total RNA from cultured cells with TRIzol Reagent (Thermo Fisher Scientific, MA, USA) following the manufacturer's instructions. For qRT-PCR detection, the reaction was conducted in ABI StepOnePlusTM real-time PCR system (Applied Biosystems, CA, USA) and GAPDH was served as the internal control. The used primers were listed in Table 1.

### Statistical analysis

GraphPad Prism 5.0 software was utilized to perform all the experiments. Results were displayed as the mean±SD and analyzed using the two-tailed Student t-test. *P* <0.05, the difference was significant. *P < 0.05, **P < 0.01, ***P < 0.001.

## Results

### Microarray data information and DEGs analysis in gastric cancer

**Figure 1:**
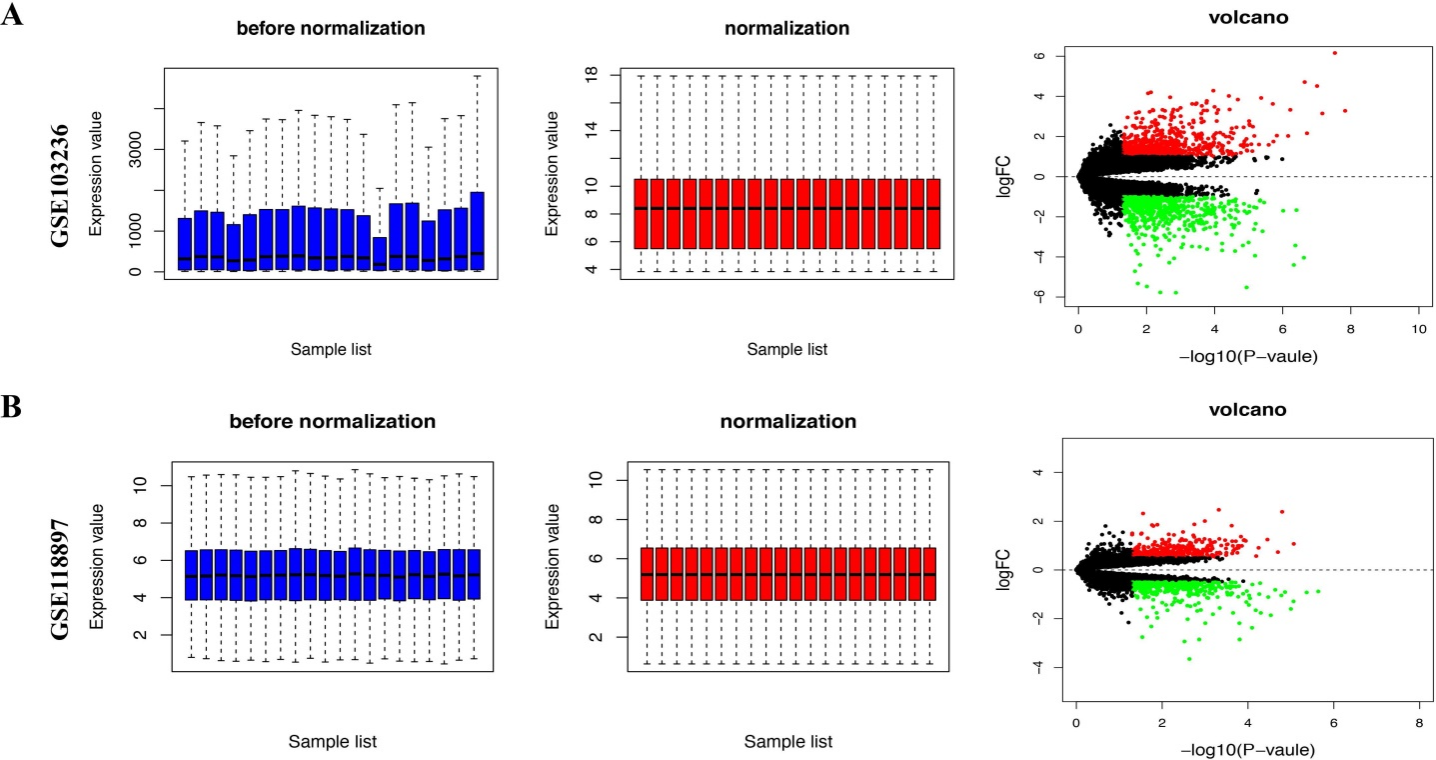
** Differential gene expression from two datasets based on GEO database.** A, The standardization (left and middle) and volcano plots (right) of GSE103236 data. B, The standardization (left and middle) and volcano plots (right) of GSE118897 data. The data before normalization were displayed as the blue bar, while the normalized data were shown as the red bar. The red and green points respectively represented upregulated and downregulated genes screened on the basis of |fold change (FC)|>2.0 and a corrected *P*-value < 0.05. Genes with no significant difference were shown as the black points.

**Figure 2:**
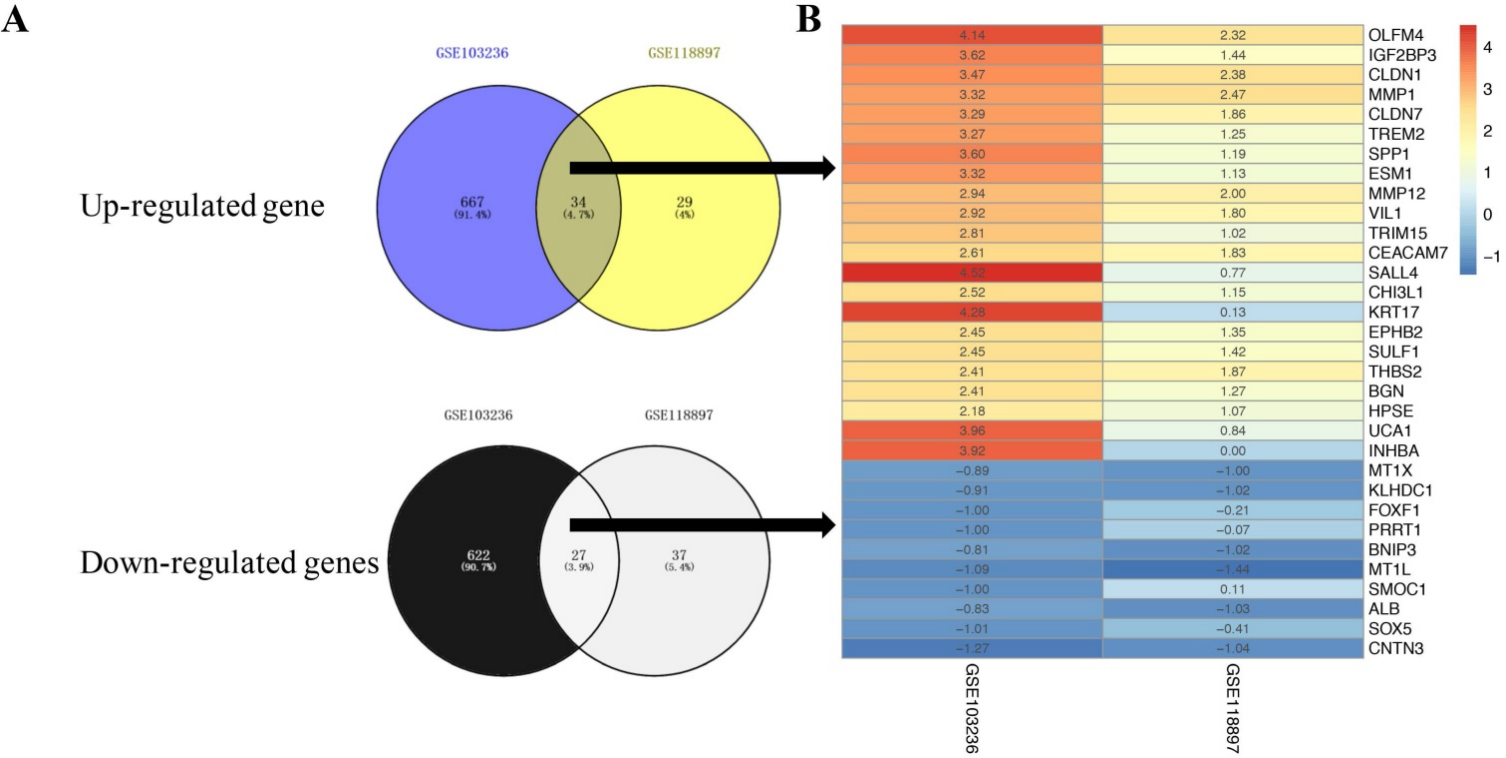
** LogFC heatmap of the image data of each expression microarray. A,** Venny diagram of intersections of up- and downregulated genes between GSE103236 data and GSE118897 data. **B,** The abscissa was defined as GEO ID, and the ordinate was defined as the gene name. Red represents logFC >0, blue represents logFC< 0, and the values in the box represent the logFC values.

We downloaded the expression microarray datasets, including GSE103236 and GSE118897, associated with gastric cancer and normalized (Figure 1A and B, left and middle). Using the limma package (|Log FC|> 1 and FDR< 0.05), we screened the two datasets to obtain DEGs. Volcano plots displayed the differential expression of multiple genes from the two sets of each sample data (Figure 1A and B, right). Overall, we obtained 1350 DEGs from GSE103236 dataset and 127 DEGs from GSE54388 dataset (Figure 2A). Venny diagram showed that there were 34 upregulated genes and 27 downregulated genes in common, respectively (Figure 2A). We used R-heatmap software to draw a heatmap of the top 32 up- and downregulated genes (Figure 2B).

### GO terms and KEGG pathway analysis

**Figure 3:**
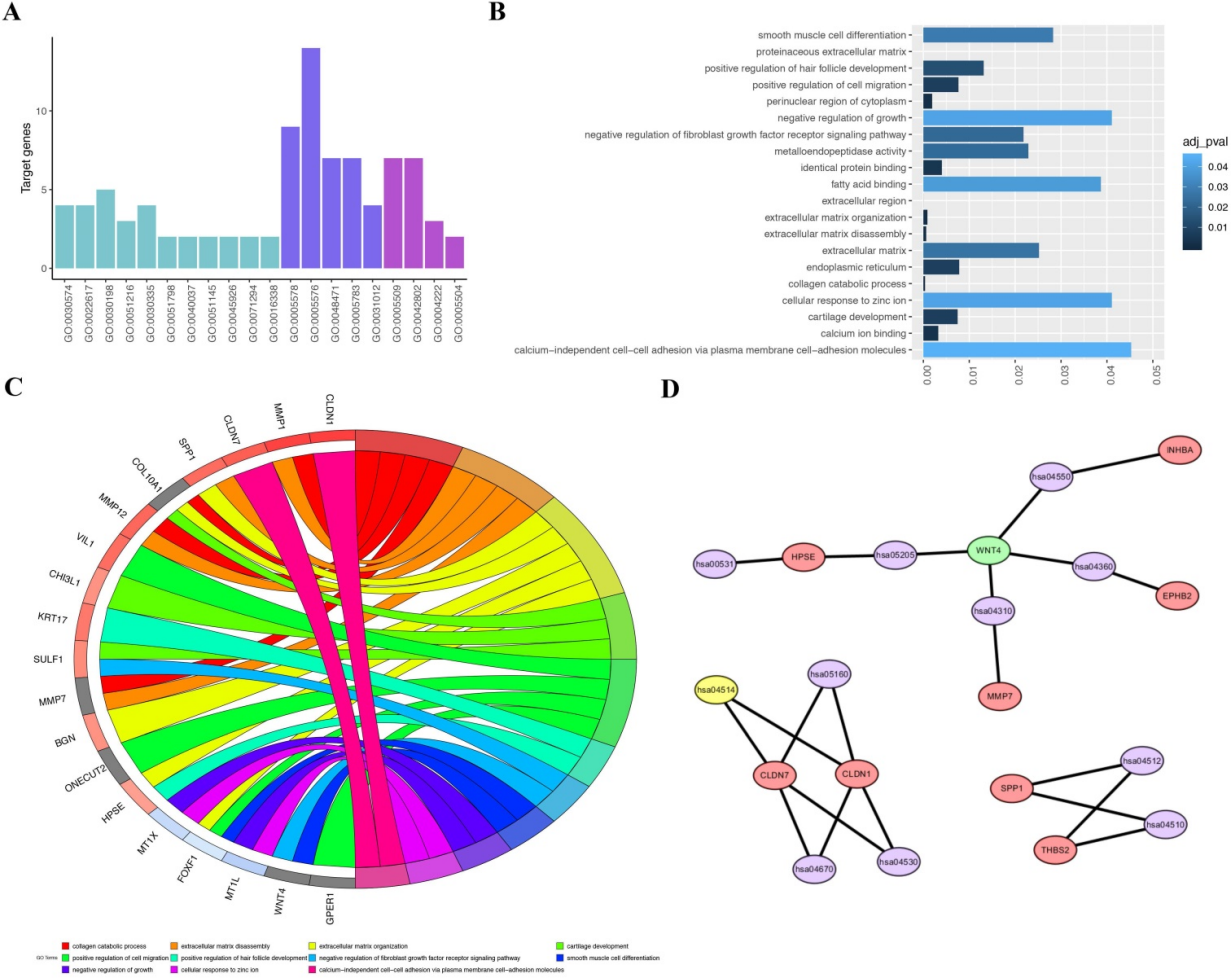
** GO terms and KEGG pathway for common DEGs.** A, GO analysis divided DEGs into three functional groups: cell composition, molecular function and biological processes. B, GO enrichment significance items of DEGs in different functional groups. C, DEGs with different GO-enriched functions were distributed in gastric cancer. D, Significant pathway enrichment of DEGs. Red represents the signaling pathway, green represents downregulated genes, purple represents signaling pathway, and yellow represents upregulated genes.

The DAVID online analysis was used to conducted biological annotation of the identified common DEGs from integrated analysis of microarray data in gastric cancer. We obtained GO functional enrichments of up- and downregulated genes with a *P*-value<0.05. Three functional groups, including molecular function, biological processes, and cell composition, were divided in GO analysis of the common DEGs (Figure 3A-C). In the molecular function group, the identified DEGs were mainly enriched in fatty acid binding, metalloendopeptidase activity, calcium ion binding and cellular response to zinc ion. In the biological process group, the common DEGs were mainly enriched in negative regulation of growth, smooth muscle cell differentiation, positive regulation of hair follicle development and negative regulation of fibroblast growth factor receptor signaling pathway. In the cell composition group, the selected DEGs were mainly enriched in extracellular matrix, endoplasmic reticulum, and cellular response to zinc ion and calcium-independent cell-cell adhesion. These results indicate that most DEGs were significantly enriched in negative regulation of growth, fatty acid binding, cellular response to zinc ion and calcium-independent cell-cell adhesion via plasma membrane cell-adhesion molecules.

Next, we used the KOBAS online analysis database (http://kobas. cbi.pku.edu.cn/) to analyze the DEGs identified from gastric cancer-integrated gene microarrays, the most significant enrichment pathway of DEGs was submitted for KEGG analysis. The signaling pathways of DEGs were mainly enriched in the Wnt signaling pathways, metabolic pathways, and pathways in cancer. The data were imported into Cytoscape to calculate the topological characteristics of the network and determine each node. The genes and pathway nodes are represented by semiellipses (Figure 3D).

### Upregulation of four key genes in stomach adenocarcinoma based on TCGA database

**Figure 4:**
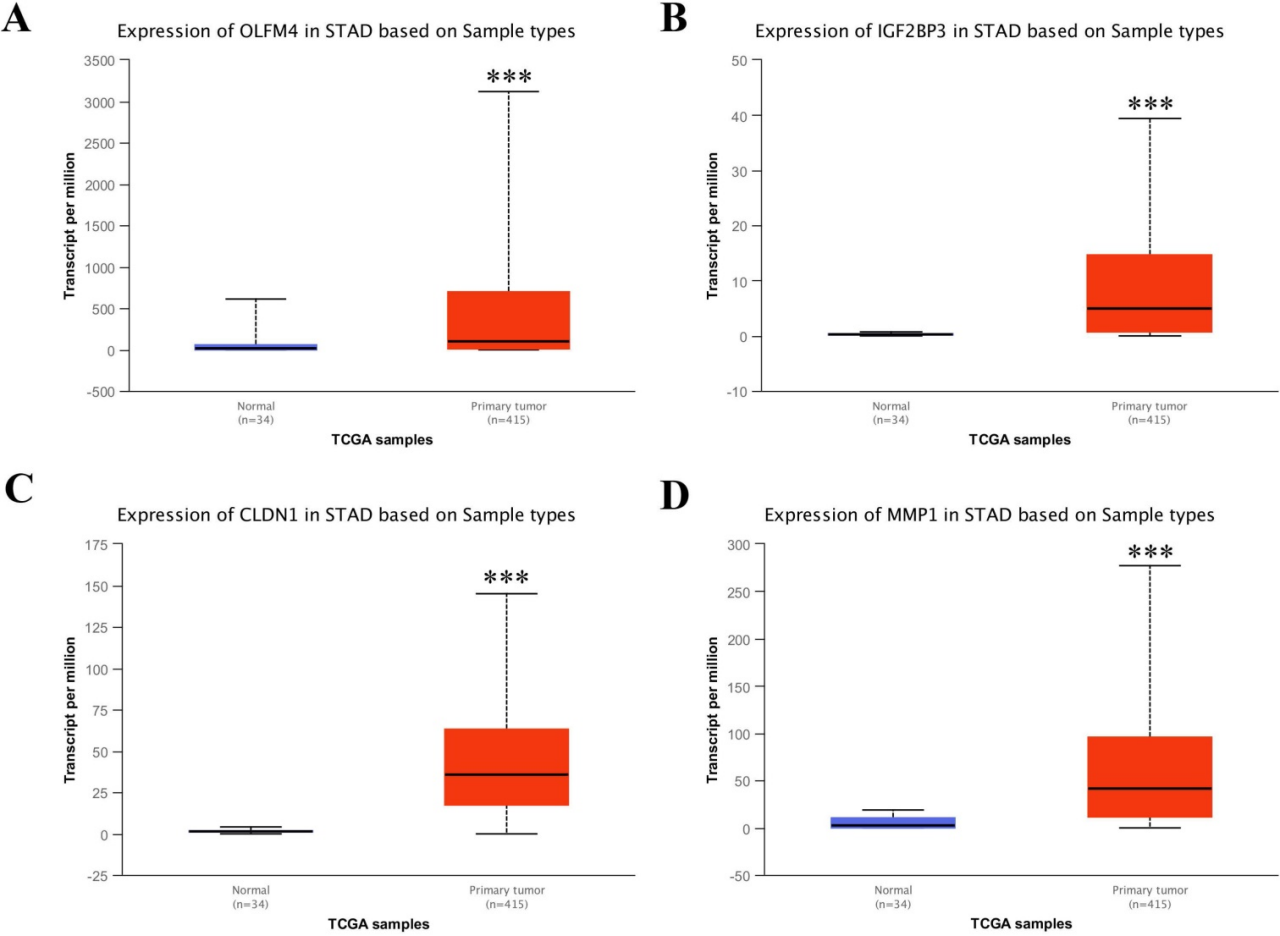
** Transcriptional levels of OLFM4, IGF2BP3, CLDN1 and MMP1 in STAD tissues and adjacent normal gastric tissues from TCGA database.** Expression panels for OLFM4 (A), IGF2BP3 (B), CLDN1 (C) and MMP1 (D) based on sample types comparing 34 normal individuals and 415 patients with STAD in TCGA database.

Based on the above analysis, we found that OLFM4, IGF2BP3, CLDN1 and MMP1 were the top 4 upregulated genes in common upregulated DEGs, which implied that they could be the candidate target for diagnostic application of GC treatment. Hence, to confirm the upregulation of these four genes in stomach adenocarcinoma (STAD), we used UALCAN web portal to detect the mRNA expressions of these four differential genes in STAD tissues compared with normal stomach tissues. The results displayed the mRNA levels of these four differential genes were dramatically upregulated in STAD tissues compared with the normal tissues (Figure 4A-D).

### Expressions of four key genes were elevated in GC cell lines and tissues

**Figure 5:**
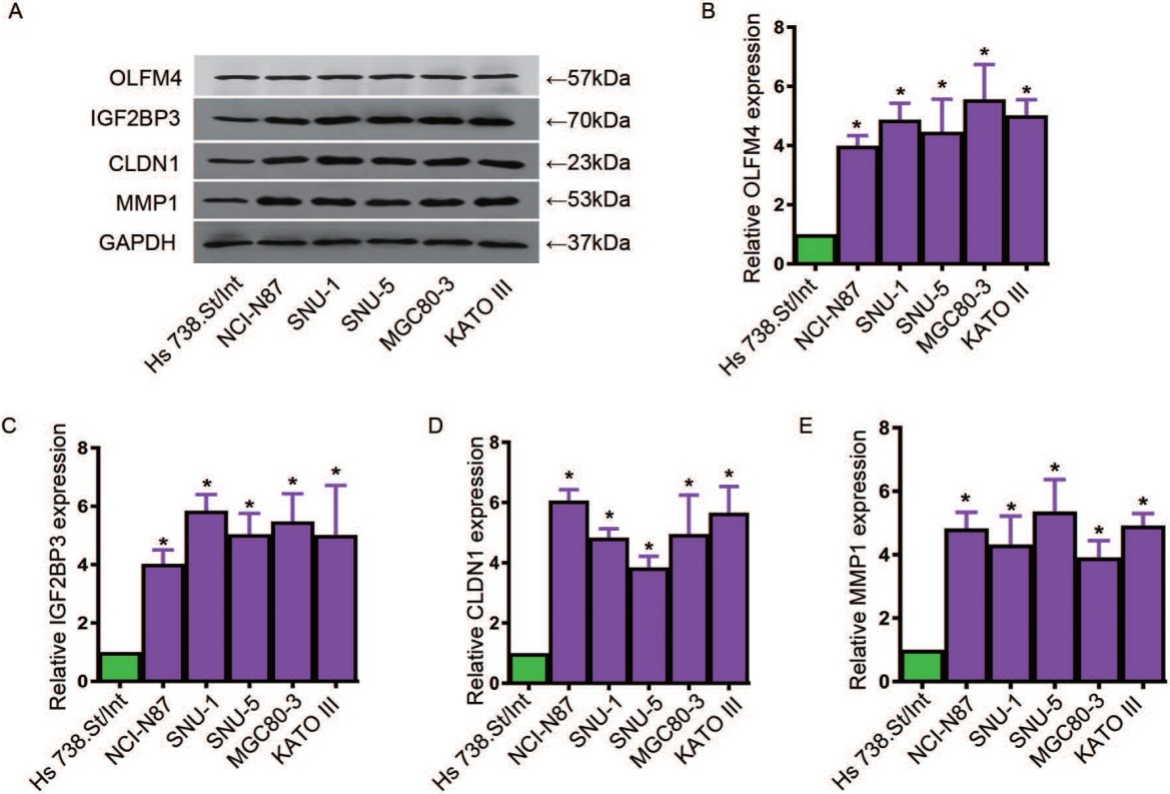
** The expressions of OLFM4, IGF2BP3, CLDN1 and MMP1 in GC cell lines.** A, western blot assay for detecting the protein levels of OLFM4, IGF2BP3, CLDN1 and MMP1 in GC cell lines. The relative mRNA levels of OLFM4 (B), IGF2BP3 (C), CLDN1 (D) and MMP1 (E) in GC cell lines.

**Figure 6:**
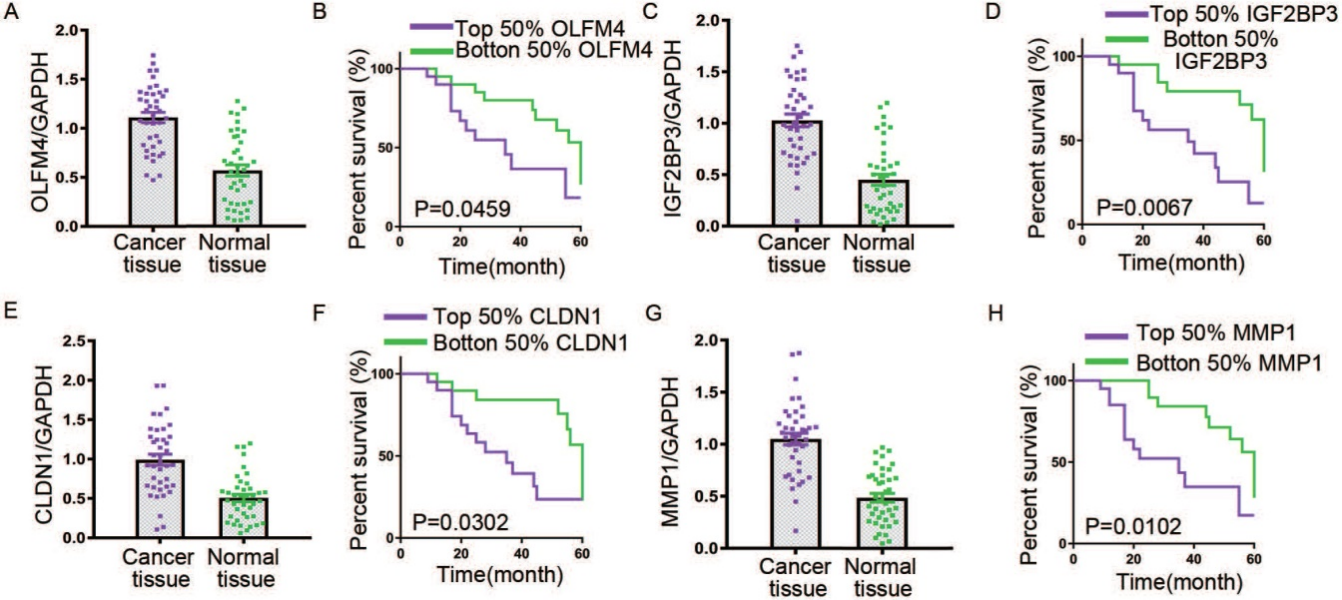
** The expressions and 5-year survival rate of OLFM4, IGF2BP3, CLDN1 and MMP1 in GC tissues.** The transcriptional levels for OLFM4 (A), IGF2BP3 (C), CLDN1 (E) and MMP1 (G) in GC tissues. The 5-year survival rate of patients with GC based on top and bottom 50% OLFM4 (B), IGF2BP3 (D), CLDN1 (F) and MMP1 (H) expression.

To further explore the expression levels of OLFM4, IGF2BP3, CLDN1 and MMP1 in GC cell lines and tissues, we used qRT-PCR assay to analyze the expressions of these genes. We cultured the GC cell lines, NCI-N87, SNU-1, MGC80-3, KATO III and SNU-5, and human fibroblast cells Hs 738.St/Int for deeply study. Western blot and qRT-PCR assay were used to detected the protein levels and mRNA levels of these four genes, respectively. Data showed that the expressions of OLFM4, IGF2BP3, CLDN1 and MMP1 in both transcription and translation levels were obviously elevated in GC cells compared with the matched normal cells (Figure 5A-E). In addition, we found that the transcriptional levels of OLFM4, IGF2BP3, CLDN1 and MMP1 were noticeably increased in GC tissues compared with the matched normal tissues (Figure 6A, C, E, G). Moreover, we explored the 5-year survival rate of patients with GC by dividing the patients into two groups based on the top and bottom 50% gene expression. Results showed that GC patients with top 50% gene expression displayed a lower 5-year survival rate compared with the patients with bottom 50% gene expression (Figure 6B, D, F, H). These results indicated that OLFM4, IGF2BP3, CLDN1 and MMP1 might be the promising potential biomarkers for diagnosis of GC.

## Discussion

Gastric cancer is one of the malignant tumors with highest mortality rate tumors worldwide 1, 2. Currently, due to complex biological processes during GC development, researchers still hard to identify the early onset of gastric cancer, which mainly contributes to the poor 5-year survival rate 3-5. Therefore, the molecular mechanism underlying carcinogenesis and development of GC is urgent to be evidenced. In past decades, microarray and high-throughput sequencing technologies have been developed well and widely used to predict potential targets for the treatment of multiple cancers by detecting the expression levels of numerous genes in humans 13-15. Even so, the pathogenic mechanism of GC still far less known with using the advanced technologies, because previous studies mostly took attention on the outcomes from a single-cohort study. Here, we integrated the gene expression profiles of GSE103236 and GSE118897 datasets downloaded from GEO database and used R software and bioinformatics to deeply analyze these datasets. We revealed 1350 DEGs from GSE103236 datasetand 127 DEGs from GSE54388 dataset. Interestingly, there were 34 upregulated genes and 27 downregulated genes in common. The top 32 most significantly up-and downregulated genes were listed, and among them, OLFM4, IGF2BP3, CLDN1 and MMP1 were the most upregulated genes. In addition, the common differential genes were divided into molecular function, biological process, and cellular component groups using GO functional annotation. GO terms analysis displayed that DEGs were mostly enriched in negative regulation of growth, fatty acid binding, cellular response to zinc ion and calcium-independent cell-cell adhesion via plasma membrane cell-adhesion molecules. Moreover, the enriched KEGG pathways of DEGs included the Wnt signaling pathway, metabolic pathways, and pathways in GC.

After a comprehensive analysis, we found OLFM4, IGF2BP3, CLDN1 and MMP1 were the most upregulated genes from both GSE103236 and GSE118897 datasets. Human olfactomedin 4 (OLFM4), also known as GW112, is normally expressed in bone marrow, prostate, stomach and others 17, 18. Several studies reported OLFM4 overexpression were also found in gastric biopsies infected with *Helicobacter pylori* 19, 20. IGF2BP3, known as IMP3, is a member of conserved IGF2 mRNA-binding protein family 21, 22. Accumulating evidences indicated that IGF2BP3 could be a promising biomarker in multiple cancers, such as colon cancer and GC 23. Claudin-1 (CLDN1) were the most consistently up-regulated genes in the tumors, such as GC 24. The phenotype with CLDN1 overexpression was generally identified as an independent and significant predictor of reduced post-operative survival. Matrix metalloproteinases (MMPs), an important family of metal-dependent enzymes, are responsible for the degradation of extracellular matrix components 25, 26. Molecular epidemiologic studies have shown associations between genetic polymorphisms of MMPs and cancer susceptibility, progression and prognosis 27-29. To test the mRNA levels of OLFM4, IGF2BP3, CLDN1 and MMP1, we used UALCAN web portal to certify the significant upregulation of these genes in STAD tissues compared with normal stomach tissues. In the final, western blot and qRT-PCR assay were performed to verify an elevation in the protein levels and mRNA levels of these four genes in the GC tissues and cell lines. Taken together, we analyzed two datasets from different groups using integrated bioinformatics analysis, and uncovered four most upregulated genes, OLFM4, IGF2BP3, CLDN1 and MMP1, in DEGs from both GSE103236 and GSE118897 datasets. Further investigation based on TCGA database or in GC cell lines confirmed the upregulation of these four genes. Thus, our results might provide novel insights for understanding GC pathogenic mechanism and potential biomarkers for early diagnosis of GC treatment.

## Figures and Tables

**Table 1:** Primers used for qRT-PCR

Gene	Primer	Sequence 5' to 3'
*OLFM4*	Forward	ACTGTCCGAATTGACATCATGG
	Reverse	TTCTGAGCTTCCACCAAAACTC
*IGF2BP3*	Forward	CCAAGCTAGACAAGCACTAGAC
	Reverse	GCGGCCATTTCATCAGGGA
*CLDN1*	Forward	CCTCCTGGGAGTGATAGCAAT
	Reverse	GGCAACTAAAATAGCCAGACCT
*MMP1*	Forward	CTCTGGAGTAATGTCACACCTCT
	Reverse	TGTTGGTCCACCTTTCATCTTC
*GAPDH*	Forward	CTGGGCTACACTGAGCACC
	Reverse	AAGTGGTCGTTGAGGGCAATG
